# In vitro adventitious root culture of *Withania somnifera* L.: a strategy for enhanced secondary metabolite production with therapeutic antioxidant and anti-inflammatory potential

**DOI:** 10.1038/s41598-026-44714-y

**Published:** 2026-04-11

**Authors:** Dina Mostafa Mohammed, Walla M. A. Abdelazeez, Ahmad A. Suliman, Amira Rashid Mohamed Sallam, Ahmed G. M. Sief-Eldein, Gamil R. Aboueldis

**Affiliations:** 1 Nutrition and Food Science Department, National Research Centre, Dokki, Giza, 12622 Egypt; 2 Botany and Microbiology Department, Faculty of Science, Al-Azhar University, Cairo, 11754 Egypt; 3 Horticultural Crops Technology Department, National Research Centre, Dokki, Giza, 12622 Egypt; 4 Plant Genetic Resources Department, Desert Research Center, Cairo, 11753 Egypt

**Keywords:** Anti-inflammatory activities, Antioxidant capacity, Cytotoxicity, Hep-G2, HPLC profiling, In vitro adventitious root cultures, Secondary metabolites, *Withania somnifera* L., Biochemistry, Biological techniques, Biotechnology, Microbiology, Plant sciences

## Abstract

The adventitious root culture is an effective in vitro technique for producing valuable secondary metabolites. These cultures operate independently of the shoots and quickly provide a substantial raw material. This study is the first report aimed at reducing the cost of biomass production by encouraging adventitious root development and examining the potential of secondary metabolites produced from adventitious root cultures of *Withania somnifera* L. to kill cancer cells (Hep-G2) and reduce in vitro inflammation activity by testing their ability to prevent lipoxygenase and proteinase from performing their activities. IBA concentrations were tested to induce adventitious roots from *W. somnifera* L. plantlet leaves; the optimal IBA concentration was 1.0 mg/L. In addition, increasing the number of subcultures led to an increase in biomass to 5.84-fold in the fifth subculture. The macronutrient content, antioxidant tests, flavonoids, phenolic, and HPLC analysis were assessed. Anticancer and anti-inflammatory characteristics were evaluated. The results of total phenolics and total flavonoids were 27.42 ± 2.57 and 7.21 ± 0.14 mg/g Dw, respectively, and total antioxidant activities, including DPPH and ABTS, were 13.61 ± 2.21 and 18.31 ± 1.34 mg/g Dw, respectively. HPLC analysis revealed that catechin and gallic acid were the highest compounds, with concentrations of 419.26 and 249.73 µg/g, respectively, which correlated with strong anti-inflammatory and cytotoxic activities in vitro. Moreover, the IC_50_ for cytotoxicity against Hep-G2 was 100 µg/ml. In conclusion, the in vitro* W. somnifera* L. adventitious roots have shown promising results in the synthesis of secondary metabolites. Moreover, these adventitious roots have significant nutritional value, which increases antioxidant activity, in addition to their potential application as new natural secondary metabolites.

## Introduction

Global population growth significantly influences the demand for agricultural land used to produce plant-based raw materials^1^. Furthermore, many plants are rare as agriculture expands and damages the environment because of the unplanned and continuous collection and use of plants as raw materials to manufacture secondary metabolites. At the same time, particular species have even become extinct^2^. Utilizing organ or cell cultures, which provide a different method for better secondary metabolite synthesis from source materials, is one of the many strategies used to address this problem^3,4^.

Organ cultures are a viable substitute for callus and suspension cell cultures due to their superior ability to create secondary metabolites and their genetic stability^5,6^. One type of organ culture is in vitro adventitious root cultures, which are defined as emerging from any part of the plant other than the actual plant root. These roots originate during normal growth and development or due to the reaction to wounding, nutritional deficiencies, or other environmental challenges and usually elongate from the petioles, nodes, internodes, and leaves^4^. Major bioactive chemicals of *W. somnifera* L. are primarily found in the root, while secondary elements can also be found in the plant’s fruits and aerial portions.

Widely recognized as "Indian Ginseng," *Withania somnifera* (Ashwagandha) holds a central position in traditional Indian medicine, particularly for the pharmaceutical value of its roots. Primarily found in dry tropical and subtropical climates, this Solanaceae family member is cultivated extensively for its diverse therapeutic profile, which includes adaptogenic, anti-inflammatory, and immunomodulatory properties. It is frequently employed to manage conditions ranging from anxiety and insomnia to arthritis and respiratory issues. These extensive health benefits are primarily attributed to specific bioactive steroidal lactones known as withanolides—most notably Withanolide-A and Withaferin-A—which are concentrated within the plant’s root system^7–15^.

Hairy root culture (HRC) is a widely used technique for producing active compounds on a large scale. The in vitro technique’s potential use is to initiate hairy root cultures for increased withanolide production and artificially increase the concentration of the withanolides present in the plant by using various elicitors^16^. The pharmaceutical industry dislikes using hairy roots for metabolite synthesis since *Agrobacterium rhizogenes* genetically modifies them, and they may also create specific substrates that might be harmful to humans^17,18^. Since in vitro adventitious root culture is non-transgenic, it is appropriate for organic or regulatory-sensitive industries. It is also easier to standardize and has a more straightforward regulatory approval process. Additionally, non-transgenic production methods may be preferred by specific consumer markets and certification bodies, which would increase their acceptance^4,19^.

Modern phytochemical research increasingly relies on the synergy between metabolomics and bioactivity profiling to pinpoint therapeutic compounds within medicinal flora. This integrated approach is exemplified by the work of Li et al.^20^, who successfully utilized network pharmacology and metabolic mapping to identify specific angiogenic elements in the Xuefu Zhuyu decoction. Such methodologies underscore the systemic biological impact of plant metabolites, transforming how we discover and validate the medicinal potential of complex botanical formulas. Similarly, Shi et al.^21^ employed UHPLC-Q-Orbitrap-MS to characterize antioxidant and anti-inflammatory phytochemicals from Kadsura coccinea, emphasizing the pharmacological value of leaf-derived extracts. Furthermore, comprehensive reviews like that of Luo et al.^22^ on Ruta graveolens illustrate the importance of correlating phytochemistry with therapeutic effects to support the development of plant-based bioactives. In this context, the current study aims not only to optimize biomass production from *W. somnifera* adventitious roots but also to profile their bioactive metabolites and assess their cytotoxic and anti-inflammatory potential, thereby contributing to evidence-based utilization of this important medicinal species.

Recent advancements in phytochemical research have emphasized the value of integrating metabolomics and bioactivity profiling to identify functional compounds from medicinal plants with therapeutic relevance. Therefore, in vitro adventitious root cultures are the best way to produce high biomass. They allow the adventitious roots to be completely independent of the plant and enable continuous root reproduction through root tip culture without genetically altering it by *A. rhizogenes*. This subsequently provides safe raw materials for mammalian cells to isolate bioactive compounds. In this regard, the adventitious root culture has the potential for biomass production all year round. This study aims not only to optimize biomass production from *W. somnifera* L. adventitious roots but also to profile their bioactive metabolites and assess their cytotoxic and anti-inflammatory potential, thereby contributing to evidence-based utilization of this important medicinal species.

## Methodology

### Materials

Seeds of *W. somnifera* L. variety Jawahar-20 collected from the Egyptian Gene Bank garden. The natural roots of *W. somnifera* L. were sourced from the General Moringa Scientific Cooperative Society located at the National Research Centre (NRC) in Giza, Egypt. MS medium was provided by Duchefa Biochemie (Haarlem, The Netherlands). The quantitative measurements were carried out using spectrophotometry kits acquired from the Biodiagnostic Company (Egypt). Naval American Research Unit-Egypt (NAmRU) provided hepatocellular carcinoma (Hep-G2). Additional compounds were provided by Merck compounds, located in Darmstadt, Germany.

## Methods

### Surface sterilization and culture conditions

**Fig. 1:**
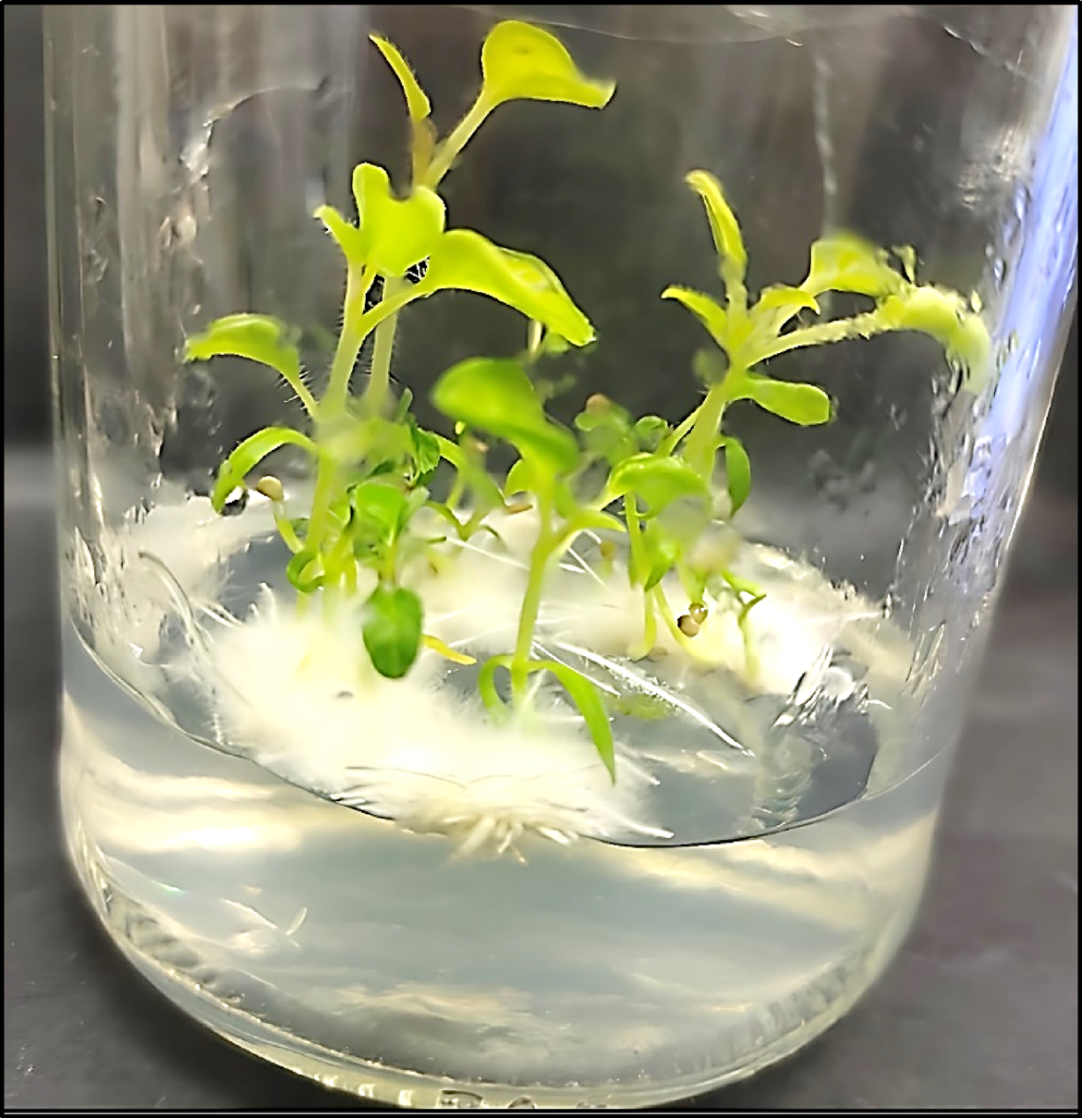
Aseptic culture of *W. somnifera* L. after 6 weeks.

To prevent contamination during growth, the seeds were surface-sterilized using commercial Clorox® (Clorox Co., Oakland, CA; 5.25% sodium hypochlorite) at a 20% (v/v) concentration for 20 min after surface sterilization, the seeds were placed on solid MS medium devoid of plant growth regulators, containing 3% (w/v) sucrose and 0.01% (w/v) myo-inositol^23^. The pH was adjusted to 5.7 ± 0.2 prior to the addition of the 2.7 g/L gelling agent (phytagel). Subsequently, all jars were incubated in darkness at 26 ± 2 °C for 1 week, followed by transfer to other incubation conditions with a photoperiod of 16/8 h (light/dark) at 26 ± 2 °C for 25 days. After 6 weeks of cultivation, plantlets reach a suitable size for the leaves to be used as explants for further experiments (Fig. 1).

### Root induction and subculturing

**Fig. 2:**
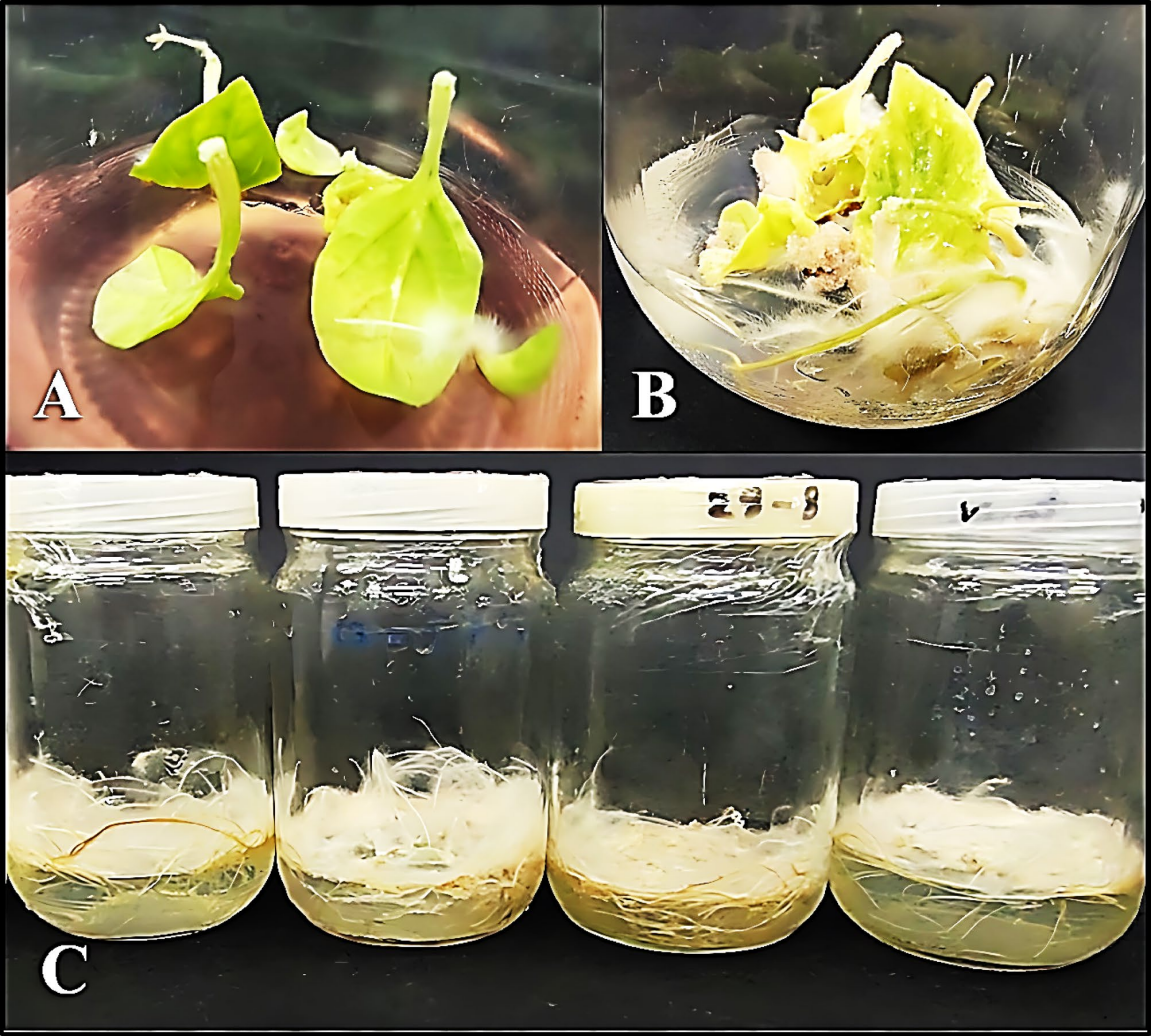
Adventitious root cultures of *W. somnifera* L. (**A**) Adventitious roots develop leaves after 2 weeks from culture on 1.0 mg/L IBA. (**B**) Adventitious roots develop after 6 weeks from cultured of explant on 1.0 mg/L IBA. (**C**) Adventitious root culture from the 5th subculture.

For induction of the adventitious root cultures, the plantlets germinating in vitro were carefully removed from jars and placed on filter paper. Using scalpels and forceps, the leaves were separated and cultivated independently on an MS solid medium enriched with varying IBA (indole-3-butyric acid) doses (0.0, 0.5, 1.0, and 2.0 mg/L). All cultured leaves were incubated at 26 ± 2 °C and a photoperiod of 16/8 h (light/dark); after 2 weeks, the adventitious roots started to develop from petioles and leaf cut places (Fig. 2A). After 6 weeks, sterile tools (scalpels and forceps) were used to cut the adventitious roots into aliquots. They were then cultivated on solid basal MS medium enhanced with 1.0 mg/L IBA and subcultured five times for a month on the exact composition^24,25^. The growth index (I) was obtained using the following formula:

$$\text{Growth index }(\mathrm{I})=\frac{\left({\mathrm{M}}_{\mathrm{max}}-{\mathrm{M}}_{0}\right)}{{\mathrm{M}}_{0}}$$

M_0_ was the adventitious root cultures’ starting mass (mg); M_max_ was the adventitious root cultures’ final mass (mg) at the conclusion of the growth cycle.

### Macronutrients analysis

2,6-Dichlorophenolindophenol (DCPIP) titration was used to quantify the amount of ascorbic acid^26^. Using the method by Suliman and Saleh^27^, the sample’s weight remained steady after being dried at 70 °C before being digested to ascertain the levels of N, P, K, and Ca in the adventitious root of *W. somnifera* L. Worsfold et al.^28^ used spectrophotometry to detect the phosphorus concentration at 650 nm. According to Cottenie et al.^29^, the potassium and calcium contents were measured using the flame photometer PFP7. The Kjeldahl Gerhard Vapodest 20 s was used to test the nitrate levels and nitrogen content^29^. Calcium and potassium chloride were used as inputs in a standard curve to produce the findings. Tests for Mn, Cu, and Zn (ppm) were conducted using the established protocols of the American Public Health Association^30^. Nevertheless, the iron (Fe) was measured by ICP (Perkin Elmer Model 4300DV). Concentrated sulfuric acid was accustomed to dehydrating the total carbohydrates, forming furfural derivatives that reacted with the anthrone reagent to generate a blue-green combination that is measurable at 620 nm^31^.

### HPLC profiling

The HPLC analysis was carried out using the Agilent 1260 range of equipment. For the separation, a 5 m, 4.6 mm × 250 mm i.d. Eclipse C18 column was utilized. At a 0.9 ml/minute flow rate, the mobile phase comprised A) water and B) a 0.05% trifluoroacetic acid solution in acetonitrile. The minutes are listed below: 0–1 (82% A), 0–9 (82% A), 0–11 (82% A), 9–11 (75% A), 11–18 (60% A), 18–22 (82% A), and 22–24 (82% A), were the consecutive times that the linear gradient was programmed into the mobile phase. It was possible to see the multi-wavelength detector at 280 nm. For every sample solution, a five μl injection volume was utilized for the sample solution. 40 °C was maintained at a steady temperature within the column. The analysis included the examination of a standard mixture consisting of all the required standards (1 mg/ml in methanol) in addition to the sample. The discovered compounds were then quantified using the peak area obtained from the standards. The phenolic peaks were identified at 280 and 320 nm wavelengths, labeled using Lab Solutions software, and quantified as micrograms per gram.

### Total phenolics, total flavonoids, and antioxidant assays

#### Total phenolics (TP)

According to Waterhouse^32^ and Mohammed et al.^33^, the total phenolic content of the dry matter was measured using a spectrophotometer. In order to conduct the test on the phenolics, which were extracted using 80% ethanol, a mixture of 1 ml of the sample and 70 ml of distilled water was used. After that, the Folin–Ciocalteau reagent and a saturated sodium carbonate solution (15 ml) were included. After 30 min at room temperature, the mixture’s absorbance at 765 nm was measured using a spectrophotometer. Gallic acid was used to create a calibration curve, per Nouman et al.^34^.

#### Total flavonoids (TF)

Soliman et al.^35^ and Pai et al.^36^ state that all flavonoid concentrations were determined using a solution of 2% AlCl_3_ in methanol. 1.5 mL of the diluted extract (1:5), 1.5 mL of AlCl_3_, and methanol were mixed. The samples were incubated at 30 °C for 10 min. We used a quercetin standard to assess the absorbance at 368 nm.

### Antioxidant assays

#### 2,2-assay for diphenyl-1-picrylhydrazyl (DPPH)

To assess the antioxidant capacity of the adventitious root extracts, a DPPH free radical scavenging assay was conducted following the protocols described by Abdelkader et al.^37^ and Brand-Williams et al.^38^. A stock solution was prepared by dissolving 24 mg/L of DPPH in 100 mL of methanol, which was then incubated in darkness for five minutes and stored at − 20 °C. For the working solution, 10 mL of this stock was diluted with 45 mL of methanol to achieve a target absorbance at 515 nm via spectrophotometry. Results were quantified using a Trolox (6-Hydroxy-2, 5, 7, 8-tetramethylchroman-2-carboxylic acid) standard curve, which exhibited linearity across a concentration range of 25 to 800 μmol. Final antioxidant activity was expressed as mg of Trolox equivalents per gram of extract (mg TE/g).

#### 2,2'-azino-bis (3-ethylbenzothiazoline-6-sulfonic acid (ABTS) assay

The ABTS + radical scavenging activity was determined following the methodology described by Abdelkader et al.^37^. To generate the ABTS + radical cation, a seven μM aqueous solution of ABTS was reacted with 2.45 mM potassium sulfate and incubated in the dark at room temperature for 16 h. Before analysis, the resulting radical solution was diluted with distilled water to achieve a stable absorbance at 734 nm (30 °C), using distilled water as a blank. For the assay, 30 μL of the sample was integrated with 3 mL of the diluted ABTS + solution, and the absorbance was recorded precisely after 6 min of reaction. Results were expressed as Trolox equivalents (TE) per 100 g of dry weight (dw), with all measurements performed in triplicate (n = 3).

### Cytotoxicity assay

The cytotoxicity of *W. somnifera* L. adventitious root culture was evaluated against Hep-G2 cancer cells using the MTT [3-(4,5-dimethylthiazol-2-yl)-2,5-diphenyltetrazolium bromide] assay to determine cell viability^39,40^. For this procedure, Hep-G2 cells were seeded into 96-well microplates at a density of 3 × 10^3^ cells/well in RPMI-1640 culture media. The experimental environment was strictly maintained at 37 °C within a 5% CO_2_ atmosphere to ensure optimal growth conditions during exposure. Staurosporine was added to the cells at different doses (0.39, 1.56, 6.25, 25, and 100 µg/ml) and adventitious root cultures of *W. somnifera* L. The cells were then cultivated for 24 h. Each well was added to a 100 µl MTT (5 mg/ml) in PBS. In order to create purple formazan crystals, the cells were kept in an incubator at 37 °C for 4 h. Once the medium had been discarded, the crystals were dissolved by adding 100 µL of DMSO to each well. An automated microplate reader was applied to measure the dissolved formazan’s optical density (OD) at 570 nm. Following the equation outlined below, the cell viability percentages were calculated:

$$\% \,{\mathrm{cell}}\,{\mathrm{viability}} = \left[ {{\mathrm{A}}_{{{\mathrm{sample}}}} /{\mathrm{A}}_{{{\mathrm{control}}}} } \right] \times 100$$

### Anti-inflammatory assays

#### Albumin denaturation assay

The albumin denaturation experiment was evaluated using the methodology described by Pieroni et al.^41^. 2 ml of adventitious root culture of *W. somnifera* L., 200 ml of fresh egg albumin, and 2.8 ml of a phosphate buffer solution with a pH of 6.4 were combined to create a reaction mixture that included 5 ml. For 15 min, the reaction mixture was incubated at 37 °C in an incubator. The temperature then increased for 5 min to 70 °C. A wavelength of 660 nm was then used to test the combination’s absorbance. The negative control was deionized water, whereas Diclofenac sodium was the positive control. In the albumin denaturation test, the expected percentage of inhibition was:

$${\mathrm{Inhibition}}\,(\% ) = 100 \times ({\mathrm{A}}_{{{\mathrm{sample}}}} /({\mathrm{A}}_{{{\mathrm{control}}}} - 1))$$

#### Proteinase inhibition assay

To assess the proteinase inhibitory activity of *W. somnifera* L. adventitious root culture, Truong et al.^42^ utilized a method where 1 ml of the culture extract (at concentrations 100, 200, 400, 600, 800, 1000, 1500, and 2000 µg/ml) was mixed with 0.06 mg of trypsin and 1 ml of pH 7.4 Tris–HCl buffer. This mixture was incubated at 37 °C for 5 min before the addition of 1 ml of 0.7% (w/v) casein protein, followed by a second 20-min incubation. The enzymatic reaction was then terminated by adding 1 ml of 70% perchloric acid. The resulting mixture was centrifuged at 1968 G for 10 min at 4 °C. Finally, the absorbance of the supernatant was measured at 210 nm using a spectrophotometer, with a buffer solution serving as the blank and indomethacin, aceclofenac, and aspirin utilized as reference compounds. The proportion of proteinase activity inhibition was determined using the following formula:

$$\% \,{\mathrm{inhibition}}\,{\mathrm{of}}\,{\mathrm{proteinase}}\,{\mathrm{activity}} = \left[ {1 - \left( {\frac{{{\mathrm{A}}2 - {\mathrm{A}}1 }}{{{\mathrm{A}}1}}} \right)} \right] \times 100$$

where A1 represents control absorbance and A2 represents sample absorbance.

#### Lipoxygenase inhibition assay

The lipoxygenase inhibitory activity of the *W. somnifera* L. adventitious root culture was evaluated using the method developed by Shrivastava et al.^43^, employing 5-lipoxygenase as the enzyme and linoleic acid as the substrate. The procedure involved mixing 10 µl of lipoxygenase (final concentration: 8000 U/ml) with 1 ml of 0.1 M sodium borate buffer (pH 8.8), and then incubating 1 ml of this enzyme solution with the root culture at concentrations 100, 250, 500, 1000, 1500, and 2000 µg/ml. The mixture was maintained at a constant temperature of 30 ± 2 °C for 5 min before the reaction was initiated by the addition of 10 µl of 10 mmol linoleic acid. Indomethacin, aceclofenac, and aspirin served as reference compounds, and the final absorbance was measured at 234 nm using a spectrophotometer to determine the level of enzyme inhibition. The formula below will be used to calculate the percentage of lipoxygenase inhibition:

$$\% \,{\mathrm{inhibition}}\,{\mathrm{of}}\,{\mathrm{proteinase}}\,{\mathrm{activity}} = \left[ {1 - \left( { \frac{{ {\mathrm{A}}2 - {\mathrm{A}}1 }}{{{\mathrm{A}}1}}} \right)} \right] \times 100$$

where A1 represents control absorbance and A2 represents sample absorbance.

### Statistical analysis

The data were shown using the mean ± SD. Five biological replicates were used for root induction and subculturing; each replicate had three explants. At the same time, the other in vitro methods were performed in three replicates. The assays under investigation were evaluated for mean and difference values using a one-way ANOVA test and Tukey post-hoc analysis (α = 0.05). The experimental data were analyzed using SPSS software (Chicago, IL, USA), with the threshold for statistical significance established at *p* ≤ 0.05.

## Results

### Establishment of in vitro adventitious root cultures

*W. somnifera* seeds surface-sterilized and cultured in vitro exhibited a germination rate and survival rate 100% without contamination using a 1.0% (commercial bleach containing 5.25% NaOCl) for 20 min. However, after 4 weeks, from the in vitro germinated plant, the leaves were separated from the stems and cultured on a basic solid MS medium supplemented with concentrations of 0.0, 0.5, 1.0, and 2.0 mg/L. Adventitious roots appeared 2 weeks after the leaves were cultured with 1.0 mg/L; meanwhile, no adventitious roots formed on the plant parts cultured on the control medium. Adventitious roots also appeared in media containing 0.5 and 2.0 mg/L indolebutyric acid (IBA) concentrations 3 weeks after culture. The results show no statistically significant difference in the percentage of adventitious root formation compared to the percentage of adventitious root formation in plant parts treated with 1.0 and 2.0 mg/L indolebutyric acid (IBA). Subsequent studies will utilize the 1 mg/L concentration, as shown in Table 1, as it is the most suitable.

**Table 1:** Effect of different concentrations of IBA on % of explant formed adventitious roots after 6 weeks and Mean number of weeks to emerge the adventitious root cultures.

IBA concentrations	% of explant formed adventitious roots after 6 weeks	Mean number of weeks to emerge the adventitious root cultures
0.0 mg/L (control)	0%^c^	0^c^
0.5 mg/L	47%^b^	3^b^
1.0 mg/L	100%^a^	2^a^
2.0 mg/L	100%^a^	3^b^

The data displays mean ± SD for n = 5. While using equivalent letters in each raw revealed no significant difference, selecting different letters produced a statistically significant change (*p* ≤ 0.05).

**Fig. 3:**
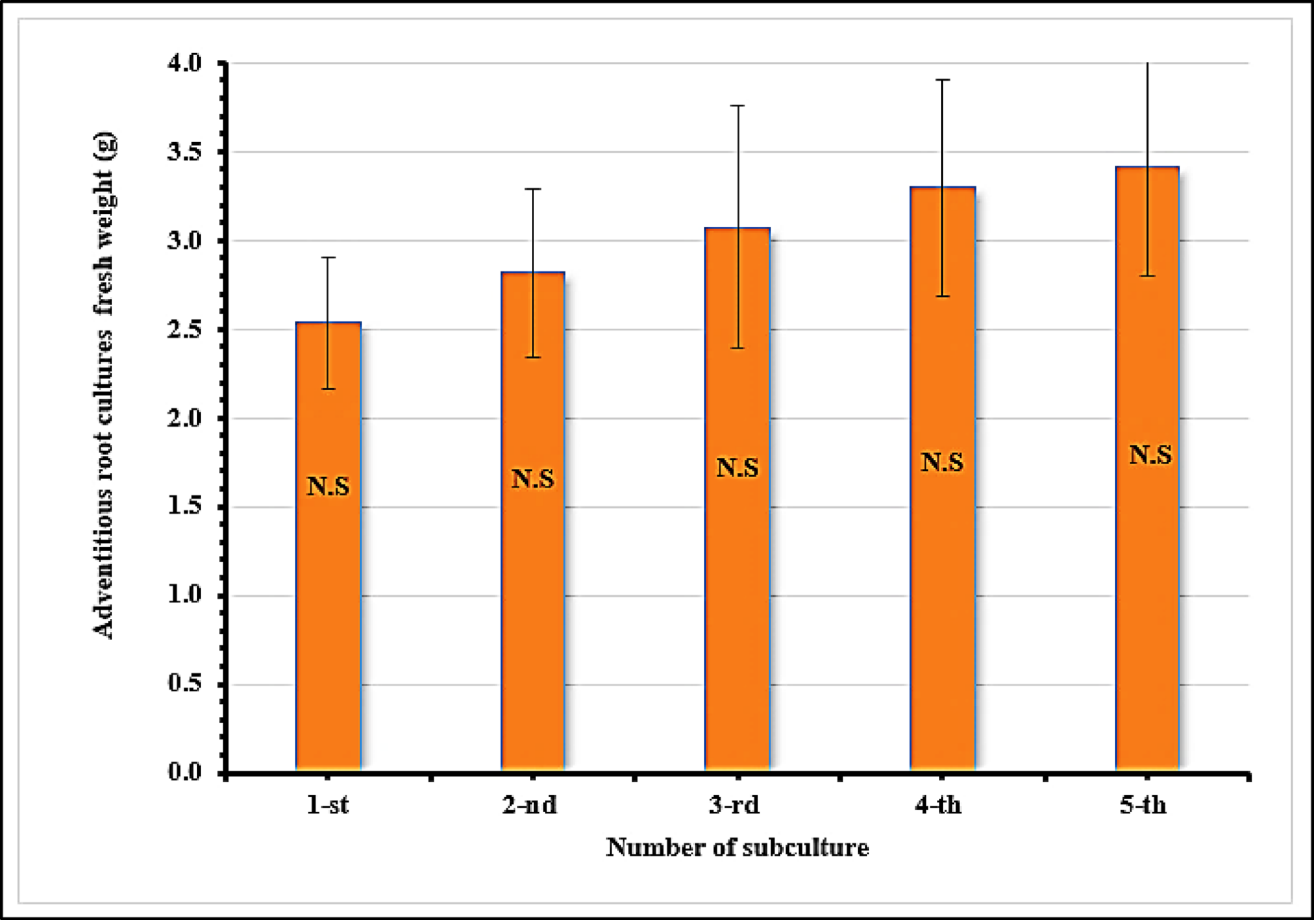
Effect of solid basal MS supplemented with 1.0 mg/L IBA on adventitious root cultures fresh weight (g/jar) of *W. somnifera* L. for five successive subculture. N.S: Not significant (*p* ≤ 0.05).

After 6 weeks, the adventitious roots that had developed were isolated from the leaves and cultivated on a basal MS medium that had been enhanced with the optimal concentration (1.0 mg/L IBA) and subcultures five times every 4 weeks on the exact composition for the subsequent trials in order to acquire sufficient adventitious root cultures (Figs. 2A–C and 3). The results indicate that the biomass increase of adventitious root cultures does not significantly change between the first and fifth subcultures, as seen in Table 2 and Fig. 3. The results were taken up to the fifth only.

**Table 2:** Effect of solid basal MS supplemented with 1.0 mg/L IBA on Growth index (I) of *W. somnifera* L. for five successive subculture.

Number of subcultures	Growth index (I)
First subculture	4.08^a^
Second subculture	4.64^a^
Third subculture	5.16^a^
Fourth subculture	5.60^a^
Fifth subculture	5.84^a^

For n = 5, the same letters mean there is no significant difference (*p* ≤ 0.05).

### Macronutrients analysis

Table 3 lists every active component identified in the adventitious root sample; it is clear how tightly these micronutrients are related to the plant’s synthesis of active chemicals. The data presented showed that the adventitious root production contained high concentrations of elements, high concentrations of vitamin C, and high concentrations of carbohydrates compared to natural roots. The vitamin C concentration in the adventitious root was 4.67 mg/g. In contrast, in the natural root, it was 2.93 mg/g, and the total carbohydrates in the adventitious root were 27.09 ml/g, whereas in the natural root, they were 24.18 ml/g. When presenting the element concentrations, the percentage of nitrogen in the adventitious root was 8.02 and for the natural root was 6.39.

**Table 3:** Macronutrients Analysis of the adventitious root cultures and natural root of *W. somnifera* L.

Macronutrients	Adventitious root culture of *W. somnifera* L.	*W. somnifera* L. natural root
Vitamin C mg/100 g	4.67 ± 1.21	2.93 ± 0.93
total carbohydrates (ml/g)	27.09 ± 3.17	24.18 ± 4.27
Total nitrogen (%)	8.06 ± 1.1	6.39 ± 1.13
Phosphorus (%)	14.3 ± 1.3	14.11 ± 1.04
Potassium (%)	34.1 ± 3.7	28.52 ± 3.81
Calcium (%)	16.3 ± 0.7	13.82 ± 1.03
Fe (ppm)	121.6 ± 0.6	109.4 ± 0.84
Mn (ppm)	51.5 ± 1.8	41.63 ± 2.82
Cu (ppm)	10.3 ± 0.4	7.96 ± 1.21
Zn (ppm)	71.3 ± 1.2	59.99 ± 3.1

In contrast, phosphorus was 14.3 for the adventitious root and 14.11 for the natural root, potassium was 34.1 for the adventitious root and 28.52 for the natural root, and calcium was 16.3 for the adventitious root and 13.82 for the natural root. As for microelements, the adventitious root content increased in all elements except copper compared to the natural root. The iron concentration was 121.6 ppm, manganese 51.5 ppm, zinc 71.3 ppm, and copper 10.3 ppm.

### HPLC profiling

**Fig. 4:**
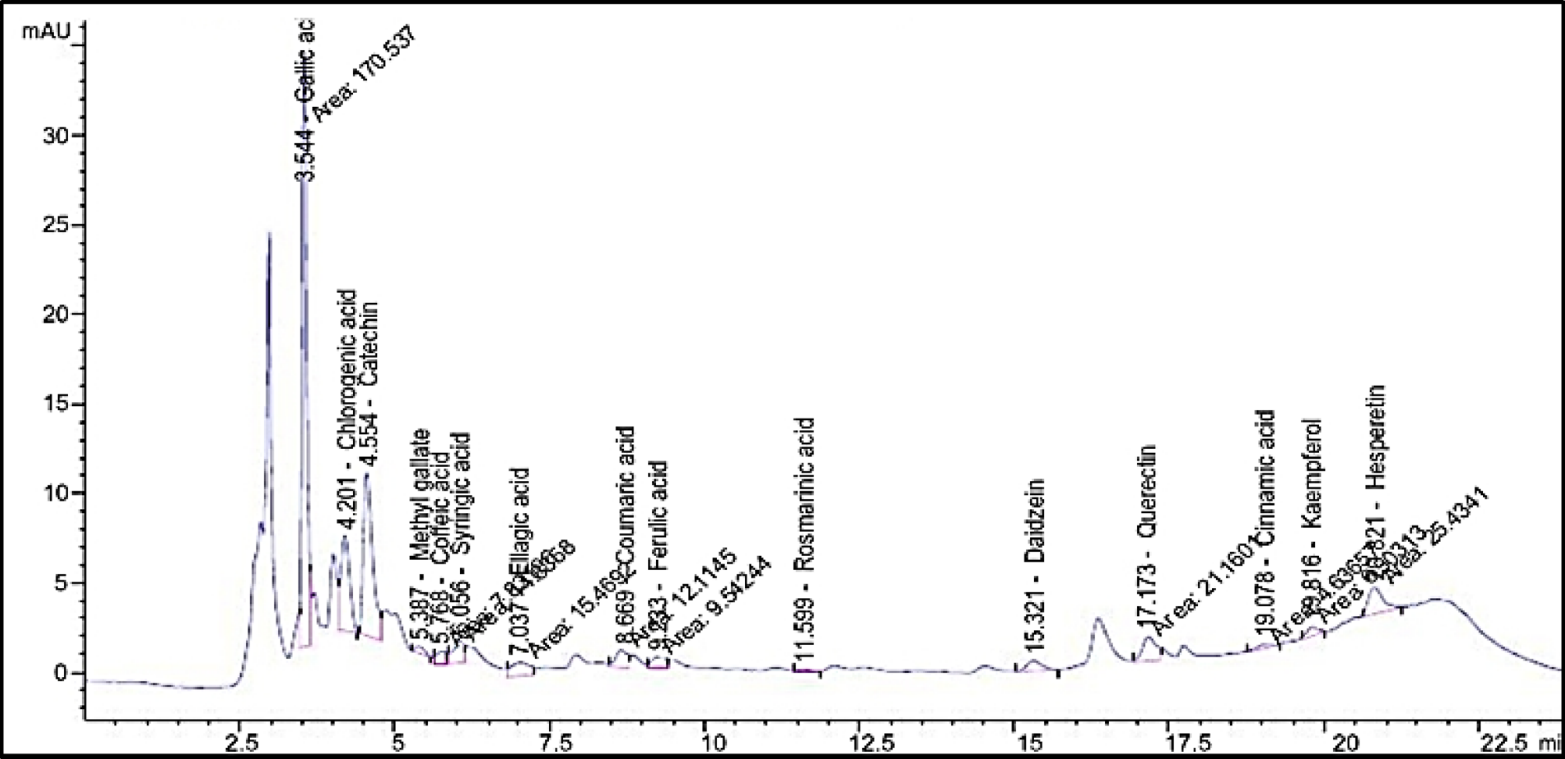
HPLC analysis of adventitious root culture of *W. somnifera* L.

The quantitative results of the HPLC profiles, detailed in Table 4 and Fig. 4, provide a comprehensive assessment of the chemical composition of the *W. somnifera* L. adventitious root culture. Through the analysis of a randomly selected sample, the investigation successfully identified and quantified the key constituents and secondary metabolites present within the extract. By comparing the retention times and peak areas against known standards, the HPLC analysis elucidated the specific active substances responsible for the culture’s biological properties, offering a precise chemical fingerprint that supports the findings of the proteinase and lipoxygenase inhibitory assays.

**Table 4:** HPLC analysis of phenolic substances from *W. somnifera* L. adventitious root cultures.

No	Compounds	Area	Conc. (µg/ml)	Conc. (µg/g)
1	Gallic acid	170.54	12.49	249.73
2	Chlorogenic acid	52.50	7.32	146.31
3	Catechin	97.58	20.96	419.26
4	Methyl gallate	3.94	0.22	4.41
5	Coffeic acid	7.83	0.40	8.04
6	Syringic acid	14.84	0.87	17.45
7	Ellagic acid	15.47	1.57	31.43
8	Coumaric acid	12.11	0.44	8.71
9	Ferulic acid	9.54	0.55	11.08
10	Rosmarinic acid	1.50	0.15	2.92
11	Daidzein	9.14	0.52	10.47
12	Querectin	21.16	2.64	52.71
13	Cinnamic acid	4.64	0.09	1.80
14	Kaempferol	6.50	0.16	3.27
15	Hesperetin	25.43	1.19	23.82

### Total phenolics, total flavonoids and antioxidant assays

The results from both the DPPH and ABTS assays demonstrated an apparent increase in antioxidant activity within the extracts, as detailed in Table 5. Correlation analysis further established a significant relationship between the specific phenolic components and the overall potency of the extracts. Specifically, the DPPH assay revealed a strong positive correlation between the concentration of phenolic components and the sample’s antioxidant capacity, suggesting that these compounds are primary contributors to the extracts’ free radical scavenging abilities.

**Table 5:** Evaluation of total phenolics, flavonoids total, and total antioxidant activities.

Antioxidant capacity	*W. somnifera* L. adventitious root cultures	*W. somnifera* L. natural root
Total Phenolics (mg/g Dw)	27.42 ± 2.57	21.94 ± 3.27
Total Flavonoids (mg/g Dw)	7.21 ± 0.14	5.63 ± 1.21
DPPH (mg/g Dw)	13.61 ± 2.21	11.40 ± 1.94
ABTS (mg/g Dw)	18.31 ± 1.34	16.28 ± 1.91

The values are mean ± SD (*p* ≤ 0.05); (n = 3).

### Correlation between phenolic and flavonoid content and antioxidant activity

**Fig. 5:**
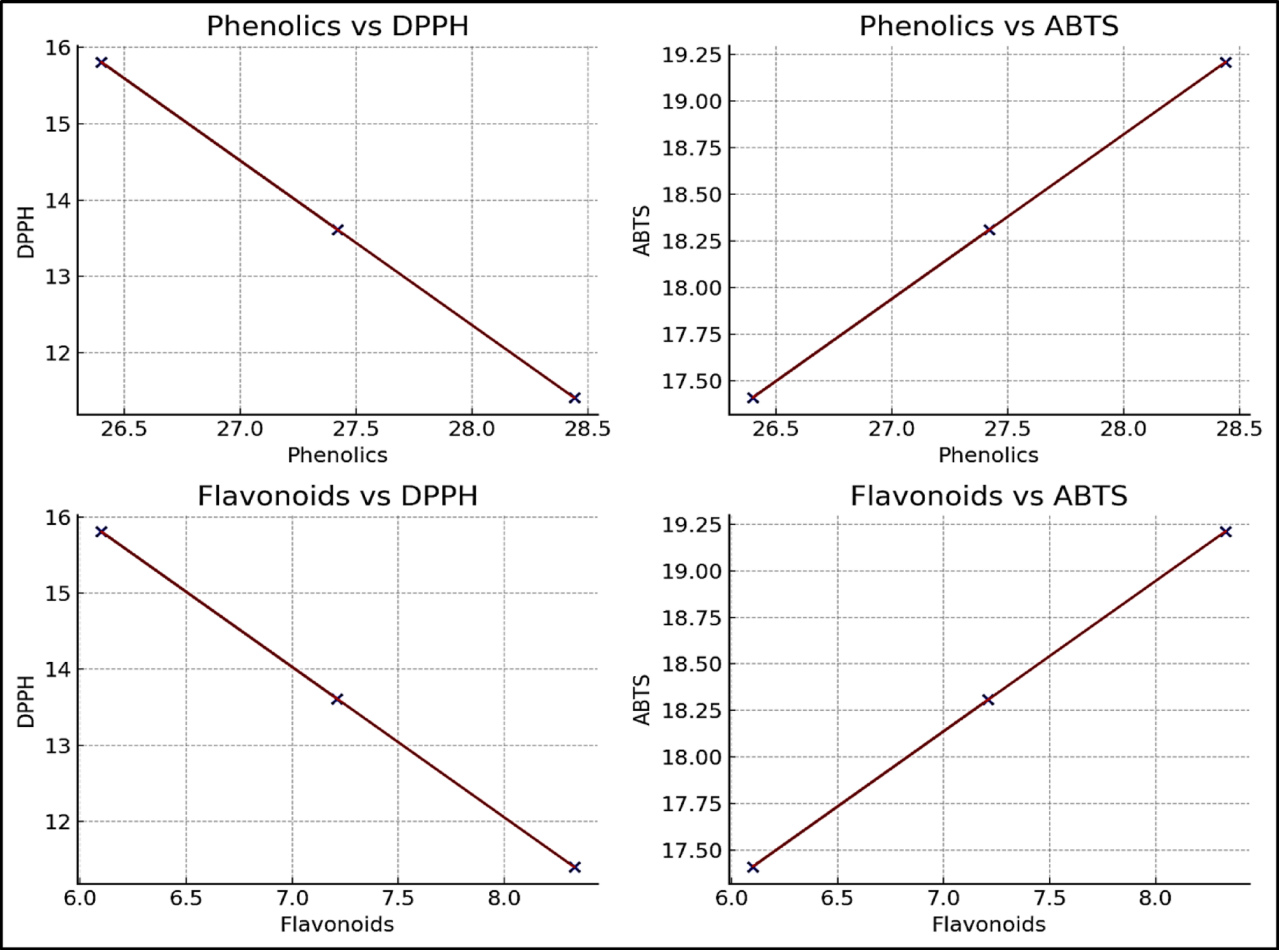
Correlation between phenolic and flavonoid content and antioxidant activity.

Correlation analysis was conducted to evaluate the relationships between total phenolic content (TP), total flavonoid content (TF), and antioxidant activity measured by DPPH and ABTS assays in *W. somnifera* L. adventitious root cultures. A strong positive correlation was observed between TP and ABTS (r = 1.000, R^2^ = 1.000, *p* < 0.00000001) and between TF and ABTS (r = 0.999997, R^2^ = 0.999993, *p* = 0.00165) (Table 6 and Fig. 5). In contrast, TP and TF exhibited strong negative correlations with DPPH activity (r = –1.000, R^2^ = 1.000, *p* < 0.0001; and r = –0.999997, R^2^ = 0.999993, *p* = 0.00165, respectively). The findings represented in Table 6 and Fig. 5 suggest that higher phenolic and flavonoid contents are closely associated with enhanced ABTS radical scavenging capacity. In contrast, their relationship with DPPH activity was inversely proportional under the tested conditions.

**Table 6:** Correlation between phenolic and flavonoid content and antioxidant activity.

Compound	Antioxidant	Pearson r	R^2^	*p* value
Phenolics	DPPH	− 1.000000	1.000000	0.00000000
Flavonoids	DPPH	− 0.999997	0.999993	0.00164821
Phenolics	ABTS	1.000000	1.000000	0.00000001
Flavonoids	ABTS	0.999997	0.999993	0.00164821

### Cytotoxicity assay

**Fig. 6:**
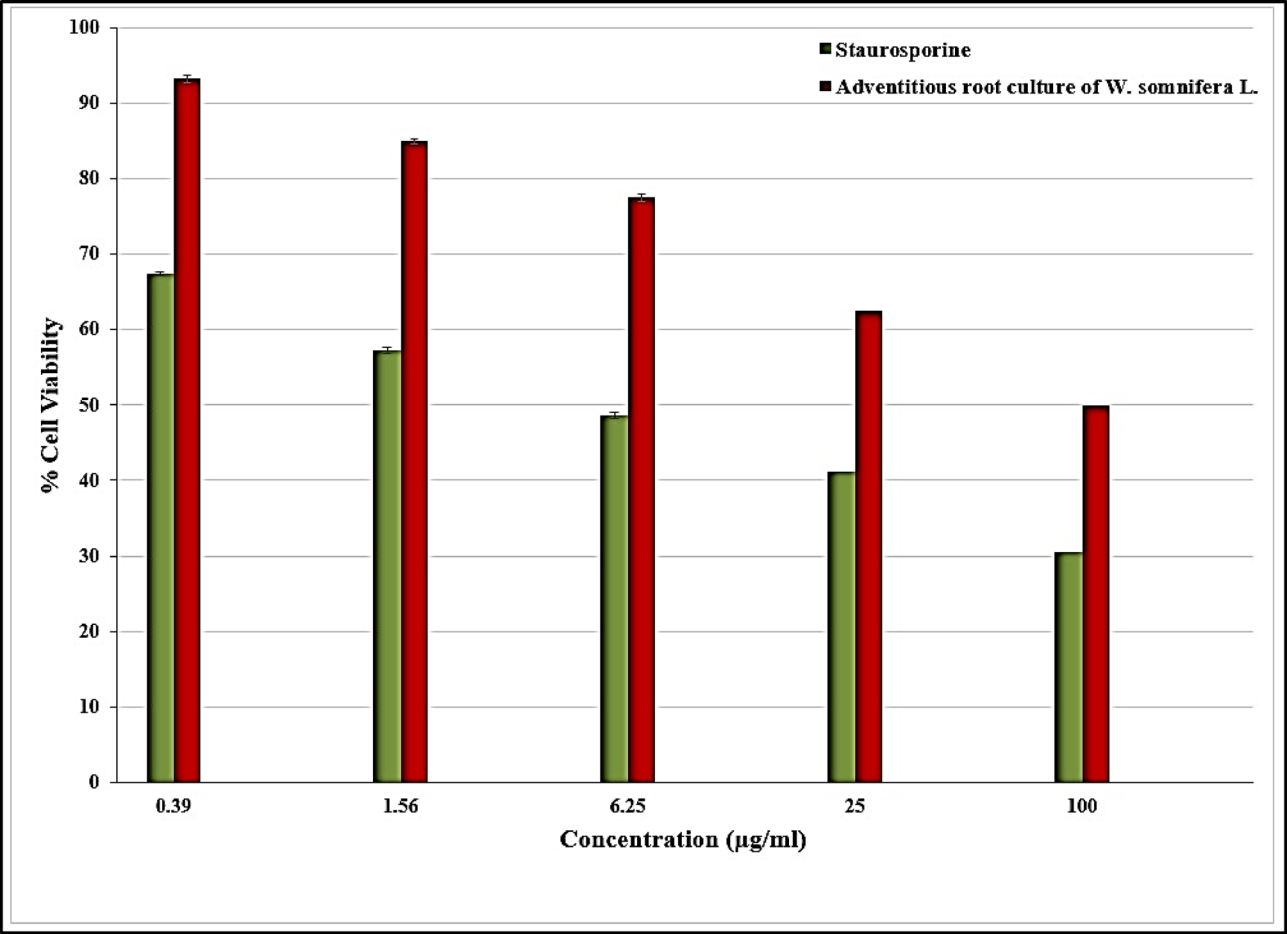
Anticancer activity of adventitious root culture of *W. somnifera* L. n = 3.

Figure 6 illustrates *the *in vitro cytotoxic activity of the *W. somnifera* L. adventitious root culture on HepG2 cell lines. Furthermore, after treatment with 1.56 µg/ml of the adventitious root culture of *W. somnifera* L., 84.95% of the hepatocellular carcinoma (HepG2) cancer cells remain healthy (Fig. 6). However, the viability percentage dropped to 50% when the concentration was raised to 100 µg/ml. For 100 µg/ml staurosporine (positive standard), the viability percentage was 30.5%. Attained 57.3% at 1.56 µg/ml. The half-maximal inhibitory concentration (IC50) was recorded at 2.67 ± 0.05 and 0.73 ± 0.02 μg/ml (for 24 h exposure) for the adventitious root culture of *W. somnifera* L. and staurosporine, respectively (Table 7). According to the data, the adventitious root culture of *W. somnifera* L. showed the most significant inhibition in the hepatocellular carcinoma cell line.

**Table 7:** IC_50_ (μg/mL) values of Adventitious root culture of *W. somnifera* L. and Staurosporine.

Cell type	IC_50_	
	Adventitious root culture of *W. somnifera* L. (µg/ml)	Staurosporine (µg/ml)
HepG2	2.67 ± 0.05	0.73 ± 0.02

The values are the IC_50_ values (*p* ≤ 0.05); (n = 3).

### Anti-inflammatory assays

#### Albumin denaturation assay

**Fig. 7:**
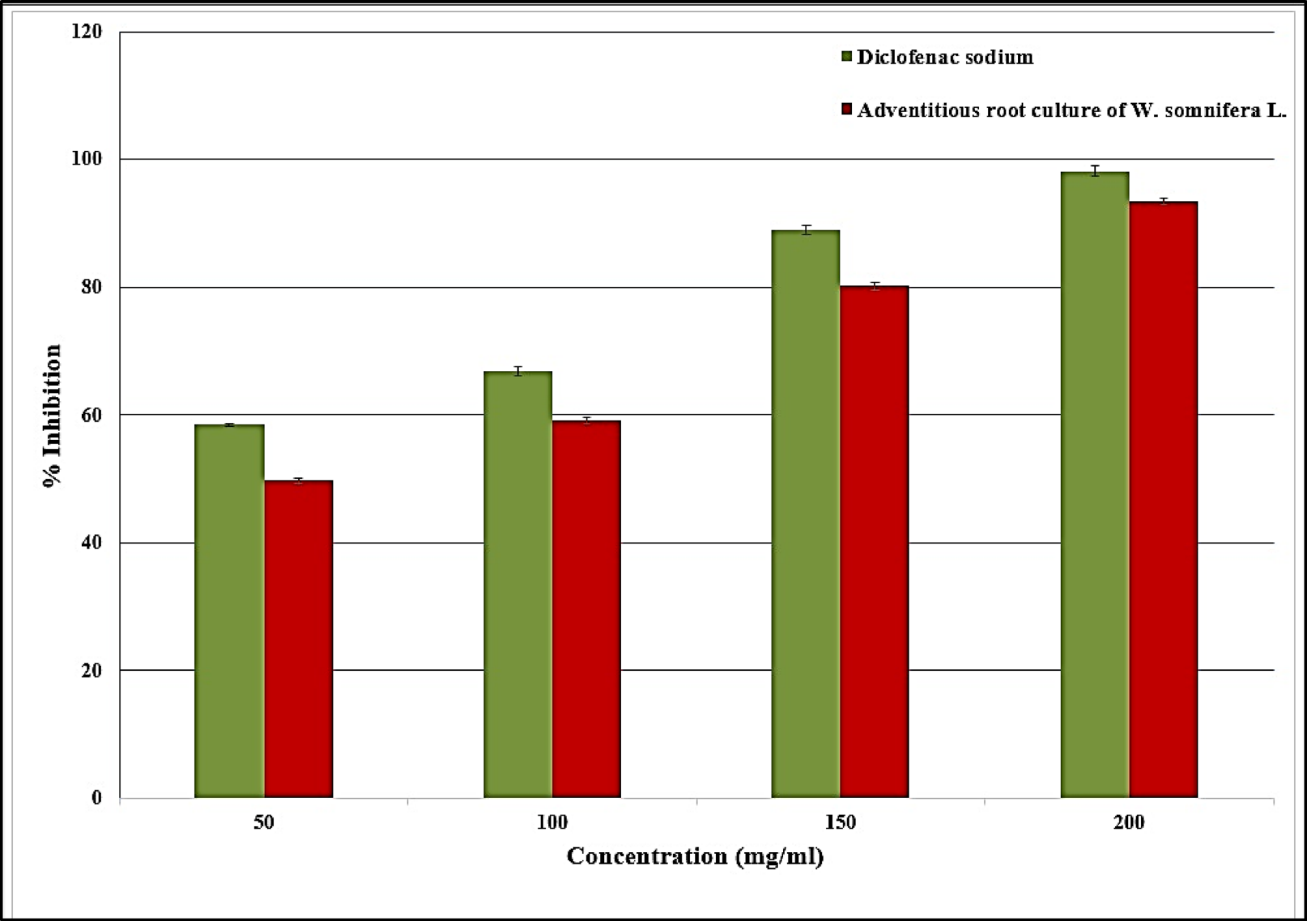
Albumin denaturation assay. n = 3.

This study evaluated the anti-inflammatory potential of the *W. somnifera* L. adventitious root culture by comparing its efficacy against the standard reference drug, diclofenac sodium, through an albumin denaturation inhibition assay. The results demonstrated that diclofenac sodium exhibited a statistically significant stability range (*p* ≤ 0.05), with inhibition percentages spanning from 58.49 ± 0.19 to 98.2 ± 0.73%. As illustrated in Fig. 7, a clear dose-dependent relationship was observed, where the proportion of albumin denaturation inhibition increased in direct correlation with the concentration of the tested samples. Specifically, the *W. somnifera* root culture showed robust inhibitory activity, with percentages ranging from 49.8 ± 0.41 at the lowest concentration to 93.5 ± 0.5 at the highest, indicating that the adventitious roots possess potent protein-stabilizing properties comparable to traditional non-steroidal anti-inflammatory drugs (NSAIDs).

#### Proteinase inhibition and lipoxygenase inhibition assays

The experimental results presented in Table 8 highlight the inhibitory potency of the *W. somnifera* L. adventitious root culture against two key inflammatory enzymes, expressed through their half-maximal inhibitory concentrations (IC_50_). The culture exhibited a robust proteinase inhibitory activity with an IC_50_ value of 30.15 ± 0.86 µg/ml. Furthermore, the evaluation of lipoxygenase inhibitory activity revealed an IC_50_ value of 40.88 ± 0.7 µg/ml. These values indicate that the root culture is more effective at inhibiting proteinase than lipoxygenase, as a lower IC_50_ signifies a higher inhibitory potency at a lower concentration.

**Table 8:** Proteinase and Lipoxygenase Inhibitions of adventitious root culture of *W. somnifera* L.

Materials	Proteinase inhibition	Lipoxygenase Inhibition
Aspirin	16.18 ± 0.67	17.95 ± 0.58
Aceclofenac	19.22 ± 0.5	12.85 ± 0.42
Indomethacin	9.1 ± 0.3	8.75 ± 0.48
Adventitious root culture of *W. somnifera* L	30.15 ± 0.86	40.88 ± 0.7

The values are the IC_50_ values (*p* ≤ 0.05); (n = 3).

## Discussion

### Root culture optimization and biomass enhancement

The establishment of adventitious root cultures is a sophisticated, multi-stage developmental process that requires a complex interplay of hormonal signals and specialized environmental factors. Central to this process is the role of auxins, which are essential for rhizogenesis; these regulators activate cells at wounding sites to initiate the formation of meristemoids, which eventually differentiate into adventitious roots^4,44^. Their internal chemical composition dictates the response of in vitro-produced explants to these signals, the application of external plant growth regulators, and various other growth-promoting elements.

In recent years, systems utilizing leaf explants have gained significant prevalence due to their superior regenerative capacity and expansive surface area, which facilitates optimal hormone absorption^4^. The inherent plasticity of leaf cells allows them to undergo rapid dedifferentiation and subsequent root development when exposed to auxin treatments. Furthermore, the characteristically lower lignin content and minimal secondary growth found in leaf tissue lower physical resistance, enabling emerging roots to penetrate the tissue more effectively and ensuring a more reliable induction process^4,45–47^.

Praveen and Murthy^14^ and Adil et al.^48^ found that IBA was effective in stimulating adventitious root growth from the leaves of *W. somnifera*. Indole butyric acid (IBA) is a crucial plant hormone, belonging to the auxin group, that stimulates root formation by increasing enzyme activity and binding to specific receptors in plant cells. It plays a crucial role in regulating gene activity and cellular processes^49,50^. Therefore, IBA was chosen to study the effect of different concentrations on stimulating the formation of adventitious roots to achieve the highest biomass production.

In the presence of exogenous IBA, the formation of adventitious roots is stimulated. Concentrations higher and lower than 1 mg/L IBA were less efficient. This explains that a balance must be struck between the internal concentration of plant hormones within the used explant and the external hormonal additions in the medium. In our study, a concentration of 1 mg/L achieved this balance.

The initial in vitro adventitious root cultures biomass was 0.5 g/jar for all subcultures. In the first subculture, the adventitious root cultures increased 4.08-fold from the initial biomass, 4.64-fold for the second subculture, 5.16-fold for the third subculture, 5.6-fold for the fourth subculture and 5.84-fold for the fifth subculture. The 5.84-fold biomass increase in subcultures indicates a sustainable platform for biomass production^51^, surpassing the 4.2-fold increase reported by Baque et al.^52^ as well as the 5-fold increase reported by Muthoharoh et al.^53^. Sub-culturing techniques can be used to maintain and enhance the characteristics of adventitious roots and obtain high yields. In this study, the subcultures contributed to the development and weight rise of adventitious root cultures.

Additionally, sub-culturing up to five subcultures progressively boosted the fresh biomass of adventitious root cultures. This is consistent with the methods that Abdelazeez et al.^5^ used. In vitro, adventitious roots can grow directly from cambium cells during organogenesis or indirectly from callus tissues due to mechanical trauma. A rapid pace of growth and an active metabolism are characteristics of adventitious root culture^54^. The use of subculture in adventitious root cultures is a common practice. The subculture cycle is one of the significant factors affecting biomass production from in vitro adventitious root culture approaches^51^.

Additionally, the subculture is a crucial procedure that allows cultural techniques to consider the frequency of subculture performances^55^. In order to maintain culture for long periods and in a healthy state, subcultured after 35 days of inoculation, the adventitious root became yellowish, indicating that the cells have entered the stationary phase, which indicates that the adventitious roots should be changed to a new medium before this time. This is consistent with the recommendation that subculture should be performed every 4–6 weeks.

### Macronutrients analysis

Nitrogen (N), Phosphorus (P), and Potassium (K)—collectively known as NPK—serve as the primary macronutrients required to sustain the physiological architecture and metabolic vigor of plant life. Nitrogen is the fundamental engine for vegetative growth, acting as a core component of chlorophyll and proteins. At the same time, phosphorus facilitates the transfer of energy and the robust development of root systems. Potassium completes this essential trio by regulating water movement and activating enzymes that bolster the plant’s natural resilience against environmental stressors. These elements do more than facilitate growth; they act as primary catalysts for secondary metabolism, the process by which plants produce bioactive compounds like alkaloids, flavonoids, and essential oils. When these nutrients are balanced within the soil, they optimize the internal synthesis of complex organic compounds, ensuring the plant reaches its full structural and biological potential^56^.

While Ashwagandha’s primary health effects are due to its phytochemicals, minerals like iron, zinc, and magnesium play a supportive and synergistic role in enhancing its benefits, especially in improving energy, reducing stress, strengthening the immune system and encouraging general health^57^. Moreover, the high mineral content is also necessary and efficient for the plant’s synthesis of active chemicals, which are necessary to complete vital tasks inside the cell^37,58^.

### HPLC profiling

Many fruits, herbs, and nuts can be used to extract gallic acid (GA), a naturally occurring secondary metabolite. Other names for it include chlorogenic acid and 3,4,5-trihydroxybenzoic acid^59^. In recent years, gallic acid’s strong anti-inflammatory qualities have attracted more interest. Gallic acid is abundant in plants, but its poor extraction rate restricts its application in development. Gallic acid may be produced chemically and biologically in vast quantities and can also be obtained from various plants. It has long been recognized that the chemical composition of *W. somnifera* L. and plant tissue offers many health benefits. According to pharmacological research, this plant contains gallic acid, which is quickly absorbed and excreted when eaten orally^60,61^. By postponing, preventing, or halting the oxidation of other compounds, ellagic acid reduces oxidative stress and traps free radicals.

Additionally, ellagic acid is an ingredient in several commercial goods with antioxidant qualities. These chemicals’ anti-mutagenic, antibacterial, antioxidant, and HIV-inhibiting properties provide them with several benefits^62,63^. Rosmarinic acid is a phenolic compound in plants that significantly increases their defenses and development. Moreover, it has antibacterial, anti-inflammatory, anticancer, anti-angiogenic, and antioxidant qualities. Certain kinds of cancer may be prevented by reducing the harm that free radicals cause to cells^39^.

Moreover, the investigation revealed the constituents and active substances. Significant biological activities are displayed by a number of important substances, especially in the areas of antioxidant and anti-inflammatory actions. A flavonoid called quercetin is well-known for its capacity to block inflammatory enzymes and cytokines, which may have therapeutic advantages for a number of inflammatory diseases. It also exhibits cardiovascular and anticancer properties. Rutin, another flavonoid, has demonstrated efficacy in lowering chronic inflammation. Additionally, in vitro investigations have demonstrated the anti-inflammatory qualities of lupeol and β-sitosterol, which were extracted from *A. myriophylla*^64^.

### Total phenolics, total flavonoids and antioxidant assays

Flavonoids are the most common type of polyphenols present in vegetables and fruits. Based on the analysis, adventitious root contains a high percentage of flavonoids and phenols. Flavonoids and phenols have potent antioxidant properties, according to Mohamed et al.^39^ and Soliman et al.^35^. These qualities may be shown by chelating minerals, scavenging reactive oxygen species (ROS) and free radicals, and stopping the oxidation of low-density lipoproteins (LDL). When the body’s ability to produce reactive oxygen species (ROS) and its antioxidant defenses are negatively disrupted, oxidative stress results. This imbalance is associated with several disorders and can cause cellular damage. In contrast, antioxidants are compounds capable of stopping or slowing harm brought on by free radicals^65^.

Thus, research on *W. somnifera* L. demonstrates that the plant contains significant concentrations of flavonoids (7.21) and total phenolics (27.42), which are effective antioxidants that can prevent free radicals from causing oxidative stress. Further research, especially in the field of pharmacokinetics, could benefit from these findings^66,67^.

### Cytotoxicity assay

The capacity of a cell to regain or sustain a state of living is assessed by a viability test. Using varying dosages of the adventitious root culture of *W. somnifera* L., this section examined the capacity of hepatocellular carcinoma (HepG2) cancer cells to recover after therapy. Staurosporine is a positive standard often employed in antitumor treatment because of its anticancer action in terms of its half-maximal inhibitory concentration (IC_50_).

According to the current research, the HepG2 cancer cell line was most susceptible to the anticancer effects of *W. somnifera* L. adventitious root culture. The profile of flavonoid compounds in the HPLC chromatography section noted the high concentration of flavonol molecules, which may be the source of this (Table 6 and Fig. 5). Flavonols, the most important flavonoid subclass, are substances in *W. somnifera* L. adventitious root culture. Biochemical properties of flavonols, including hepatoprotective, cardiovascular, and anti-inflammatory actions, have been shown. Additionally, *W. somnifera* L. adventitious root culture secondary metabolites have shown potent anti-inflammatory and anti-carcinogenic qualities^24,68^.

Numerous flavonoids and phenols are known to have potent anti-inflammatory effects. According to the National Institutes of Health (NIH), quercetin is one of the most studied and potent of them. Gallic acid, catechin, chlorogenic acid, hesperetin, and kaempferol are other noteworthy examples. Through a variety of mechanisms, such as blocking pro-inflammatory cytokines, enzymes like COX and LOX, and altering cellular signalling pathways, these drugs reduce inflammation^69^.

### Anti-inflammatory assays

All the bioactive components of the adventitious root culture of *W. somnifera* L., presented in Table 6, provide anti-inflammatory properties. Furthermore, there is a strong link between oxidation and inflammation since inflammation results from free radicals, which harm cells. Inflammation is a protective mechanism that organisms utilize to remove detrimental stimuli and a signal to initiate the healing process^70^.

### Albumin denaturation assay

The adventitious root culture of *W. somnifera* L. was evaluated for its anti-inflammatory properties in vitro using the albumin denaturation technique. According to Acharya and Chaudhuri^71^, denaturation alters the electrostatic, hydrogen, hydrophobic, and disulfide bonds that keep proteins in their three-dimensional structure. By producing self-antigens in vivo, this denaturation triggers the inflammatory response.

Polyphenols may account for the beneficial effects of *W. somnifera* L. adventitious root culture on inflammation^72^. Moreover, polyphenols influence many molecular targets implicated in inflammatory signaling pathways and lower indices of inflammation^73^.

### Proteinase and lipoxygenase inhibition assays

Since protease enzymes are known to be linked to inflammatory processes, the present research examined the anti-inflammatory effectiveness of inhibiting these enzymes. They can hydrolyze other proteins and break down peptide bonds. Furthermore, they may induce inflammation by regulating the synthesis and activity of chemokines, pro-inflammatory cytokines, and other immune components^74^. Using aspirin, aceclofenac, and indomethacin as reference drugs, the research investigated the proteinase inhibition of the adventitious root culture of *W. somnifera* L. (Table 8).

Furthermore, using lipoxidase as the enzyme and linoleic acid as the substrate, minor modifications were made to demonstrate the efficacy of lipoxygenase inhibition^75^. This activity was conducted because the lipoxygenase enzyme pathway plays a significant role in developing inflammatory disorders. It is well established that non-inflammatory medications promote tissue regeneration and reduce lipoxygenase activity. Arachidonic acid, linoleic acid, and linolenic acid are polyunsaturated fatty acids oxidized to form hydroperoxide, producing the single-unit enzyme called lipoxygenase (LOX). Mammals often include 5-lipoxygenase, which is produced from arachidonic acid’s 5-carbon position. Many immune, epithelial, and cancerous cells have the LOX protein. Numerous physiological conditions, including stroke, neurological illnesses, skin conditions, cardiovascular problems, and cancer, depend on it^42,76^. The adventitious root culture of *W. somnifera* L.'s bioactivity may impact its anti-inflammatory qualities. The study findings shown in Table 8 make this evident.

### Study limitations

A potential limitation of this study lies in the scalability for industrial applications, which remains a significant challenge that warrants further investigation, including:

- **Lack of in vivo validation:** A controlled environment is provided for testing a hypothesis in in vitro experiments, such as those carried out in a lab with cell cultures (often in Petri dishes). In contrast, a living body (in vivo) is far more complicated. It has many distinct cell types and tissues interacting with one another, a circulatory system for distributing substances, a metabolism for breaking things down, and an immune system for responding to them. Because of things like inadequate absorption, the liver’s quick breakdown, or unanticipated adverse effects on other organs, a medication that appears promising in a cell culture may be harmful or ineffective in vivo. Consequently, the results are preliminary and cannot be directly turned into a viable therapeutic for a living organism without in vivo validation.
- **No mechanistic assays to define mode of cytotoxicity:** When a substance is cytotoxic, it indicates it is harmful to cells. However, how is it poisonous? Does it trigger apoptosis, or programmed cell death, in the cell? Does it interfere with the mitochondria’s ability to produce energy? Does it interfere with the cell’s capacity to multiply or harm its DNA? The precise process of cell death cannot be determined without the use of specialized techniques; such as flow cytometry to examine DNA damage or Western blots to identify proteins implicated in apoptosis. This is a significant drawback as knowledge of the mechanism may help determine how a medication should be taken, what other medications it may be used with, and what adverse effects to anticipate. A drug that targets the cell membrane may be an effective antifungal agent, but one that interferes with DNA replication would be a suitable option for treating cancer.
- **Single cell line tested:** Cell lines are lab-grown, immortalized cells. Not all of them are the same. A healthy liver cell line or a colon cancer cell line will act differently from a breast cancer cell line. A compound’s possible effects are only partially revealed when it is tested on a single cell line. It would be like attempting to comprehend the operation of an automobile by only examining the engine of one particular model. The substance may be totally useless against other cell lines, but quite successful against the one tested. Additionally, it could be harmful to other cell lines, even healthy ones. To further understand the compound’s selectivity and any adverse effects, a thorough investigation would compare it to a panel of several cell lines, including both healthy cells and various cancer types.
- **No metabolic pathway analysis for withanolide biosynthesis:** Plants such as *W. somnifera* (ashwagandha) include a family of naturally occurring steroids called withanolides. Knowing how these substances are made is essential if the research is about them. Analyzing metabolic pathways entails examining the series of chemical processes that result in the formation of a molecule. Without this investigation, we would not have been able to determine whether genes, enzymes, or environmental conditions contribute to the production of these withanolides. This is a drawback as it keeps us from producing these molecules as efficiently as possible. For instance, we could genetically modify a plant to make more of the desired withanolide if we identified the important enzyme in the biosynthesis process. With this knowledge, we may also synthesize the molecule in a lab, which could be more economical and practical than obtaining it from plants.

## Conclusion and future perspective

The recent shift toward utilizing in vitro adventitious root cultures has revolutionized the production of secondary metabolites, offering a sustainable and efficient alternative to the depletion of natural plant resources. Unlike *Agrobacterium rhizogenes*-mediated hairy root systems, which risk genetic instability and unpredictable chemical fluctuations, adventitious cultures provide a stable, cost-effective, and genetically natural biomass source that ensures the consistency and efficacy of pharmaceutical raw materials. The present investigation highlights the therapeutic potential of *Withania somnifera* L. adventitious roots, demonstrating their potent antioxidant, anticancer, and anti-inflammatory properties, particularly through the significant inhibition of hepatocellular carcinoma cell lines. These bioactive components offer a promising avenue for preventing chronic conditions like cardiovascular disease and malignancy, bridging the gap between biotechnology, biodiversity, and clinical research. Looking forward, the expansion of this technology into large-scale bioreactors could further industrialize production, though future research must address practical challenges such as subculture-dependent metabolic variability and the logistics of industrial implementation.

## Data Availability

The data analyzed during the current study available from the corresponding author on reasonable request.
